# A121 QUANTIFYING INTER-OBSERVER VARIABILITY IN THE SEGMENTATION OF RECTAL TUMORS IN ENDOSCOPY IMAGES AND ITS EFFECTS ON DEEP LEARNING

**DOI:** 10.1093/jcag/gwab049.120

**Published:** 2022-02-21

**Authors:** L L Weishaupt, T Vuong, A Thibodeau-Antonacci, A Garant, K S Singh, C Miller, A Martin, S Enger

**Affiliations:** 1 Medical Physics, McGill University, Montreal, QC, Canada; 2 The University of Texas Southwestern Medical Center Department of Neuroscience, Dallas, TX; 3 Sir Mortimer B Davis Jewish General Hospital, Montreal, QC, Canada; 4 CHU de Quebec-Universite Laval, Quebec, QC, Canada

## Abstract

**Background:**

Tumor delineation in endoscopy images is a crucial part of clinical diagnoses and treatment planning for rectal cancer patients. However, it is challenging to detect and adequately determine the size of tumors in these images, especially for inexperienced clinicians. This motivates the need for a standardized, automated segmentation method. While deep learning has proven to be a powerful tool for medical image segmentation, it requires a large quantity of high-quality annotated training data. Since the annotation of endoscopy images is prone to high inter-observer variability, creating a robust unbiased deep learning model for this task is challenging.

**Aims:**

To quantify the inter-observer variability in the manual segmentation of tumors in endoscopy images of rectal cancer patients and investigate an automated approach using deep learning.

**Methods:**

Three gastrointestinal physicians and radiation oncologists (G1, G2, and G3) segmented 2833 endoscopy images into tumor and non-tumor regions. The whole image classifications and the pixelwise classifications into tumor and non-tumor were compared to quantify the inter-observer variability. Each manual annotator is from a different institution.

Three different deep learning architectures (FCN32, U-Net, and SegNet) were trained on the binary contours created by G2. This naive approach investigates the effectiveness of neglecting any information about the uncertainty associated with the task of tumor delineation.

Finally, segmentations from G2 and the deep learning models’ predictions were compared against ground truth labels from G1 and G3, and accuracy, sensitivity, specificity, precision, and F1 scores were computed for images where both segmentations contained tumors.

**Results:**

The deep-learning segmentation took less than 1 second, while manual segmentation took approximately 10 seconds per image. There was significant inter-observer variability for the whole-image classifications made by the manual annotators (Figure 1A). The segmentation scores achieved by the deep learning models (SegNet F1:0.80±0.08) were comparable to the inter-observer variability for the pixel-wise image classification (Figure 1B).

**Conclusions:**

The large inter-observer variability observed in this study indicates a need for an automated segmentation tool for tumors in endoscopy images of rectal cancer patients. While deep learning models trained on a single observer’s labels can segment tumors with an accuracy similar to the inter-observer variability, these models do not accurately reflect the intrinsic uncertainty associated with tumor delineation. In our ongoing studies, we investigate training a model with all observers’ contours to reflect the uncertainty associated with the tumor segmentations.

**Funding Agencies:**

CIHRNSERC

**Figure:**
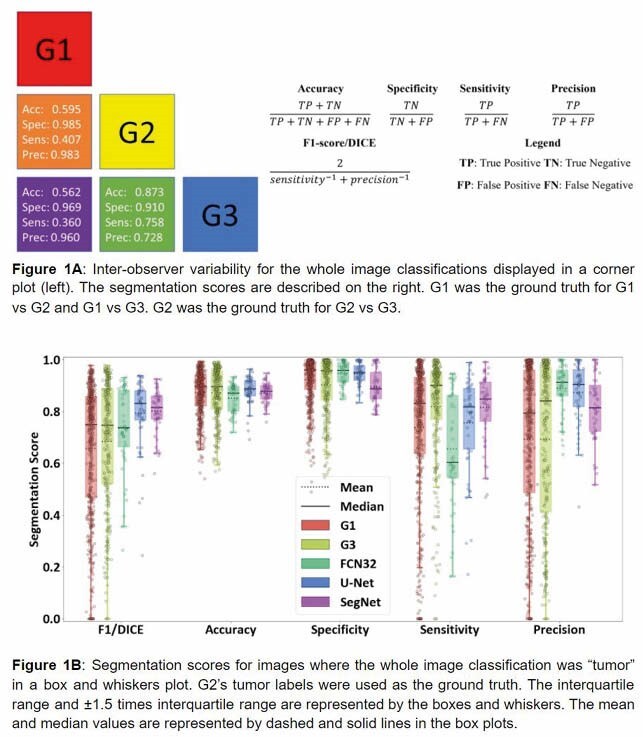
**Figure 1A:** Inter-observer variability for the whole image classifications displayed in a corner plot (left). The segmentation scores are described on the right. G1 was the ground truth for G1 vs G2 and G1 vs G3. G2 was the ground truth for G2 vs G3. **Figure 1B:** Segmentation scores for images where the whole image classification was “tumor” in a box and whiskers plot. G2’s tumor labels were used as the ground truth. The interquartile range and ±1.5 times interquartile range are represented by the boxes and whiskers. The mean and median values are represented by dashed and solid lines in the box plots.

